# The Composition and Concordance of *Lactobacillus* Populations of Infant Gut and the Corresponding Breast-Milk and Maternal Gut

**DOI:** 10.3389/fmicb.2020.597911

**Published:** 2020-12-21

**Authors:** Xuyao Zhang, Saiyidan Mushajiang, Baolong Luo, Fengwei Tian, Yongqing Ni, Wenli Yan

**Affiliations:** ^1^School of Food Science and Technology, Shihezi University, Shihezi, China; ^2^School of Food Science and Technology, Jiangnan University, Wuxi, China

**Keywords:** *Lactobacillus* populations, mother-breast milk-infant “triad, ” composition and concordance, selective media, molecular methods

## Abstract

The maternal gut is the principal source of commensal bacteria in the infant gut during the lactation stage, where breast milk acts as an intermediary for the transfer of potential probiotic bacteria consortia, including *Lactobacillus*. This study aimed to characterize the bacterial communities in human milk, maternal, and infant feces in a small yet very homogeneous cohort of 25 healthy mother–infant pairs in northwestern China (*n* = 25, infant age from 7 days to 2 years), with special emphasis on the cooccurrence and vertical transfer of *Lactobacillus* phylotypes at the species or strain level in mother-breast milk-infant triads. Accurate sequencing analysis revealed that among 73 *Lactobacillus* zero-radius operational classification units (ZOTUs) identified, 58 belonging to 18 recognized species or species groups were distributed in all three types of samples. *Lactobacillus ruminis*, *L*. *mucosae* and *L*. *gasseri*-*johnsonii* as true residents were the most represented in all three ecosystems, whereas the content of *Lactobacillus* phylotypes commonly developed as probiotics was not dominant. While the numbers of *Lactobacillus* species in breast milk and infant feces were greater than that in maternal feces, principal coordinates analysis (PCoA) based on beta diversity, coupled with the frequency of isolates determined by culture methods, showed that the *Lactobacillus* community in the infant gut was more similar to that in the maternal gut than to that in breast milk, suggesting that the gut is niche selective for *Lactobacillus* populations. In addition, identical strains of *L*. *ruminis*, *L*. *paracasei*, *L*. *mucosae* and *L*. *salivarius* were isolated from multiple mother–infant pairs, supporting the hypothesis that vertical transfer of bacteria via breastfeeding contributes to the initial establishment of the microbiota in the developing infant intestine.

## Introduction

The human gut microbiota establishment and formation in the first 1,000 days of life play an irreplaceable role in the long-term health and well-being of the host (Matamoros et al., 2013; Marchesi et al., 2016). Breastfeeding and breast milk are the major drivers of infant gut microbiota development affecting this process (Gritz and Bhandari, 2015; Fehr et al., 2020). It is well established that breast milk provides infants with nutrients necessary for growth and development, and contains lactoferrin, breast milk oligosaccharides, immune cells, regulatory cytokines, and other biologically active factors, thus creating a favorable environment for the colonization of beneficial bacteria in the gut of newborns (Walker, 2010; Jeurink et al., 2013; Mastromarino et al., 2014; Praticò et al., 2014; Cacho and Lawrence, 2017). However, it has been controversial whether milk bacteria play a role in seeding the infant gut and the origins of bacteria in human milk. To our knowledge, it is generally believed that there are two possible sources of microbes in breast milk: maternal gut bacterial translocation (entero-mammary pathway) and environmental bacterial exposure during nursing (McGuire and McGuire, 2017).

It has been suggested that contamination from maternal skin-associated species during breastfeeding contributes to the bacterial communities in human milk (Rodríguez, 2014). However, members of *Lactobacillus* were scarcely recovered in human skin specimens when using either classical plating techniques and/or DNA sequencing-based approaches, indicating that *Lactobacillus* species present in milk may not be the result of contamination from the surrounding breast skin (Pannaraj et al., 2017; Ferretti et al., 2018; Timm et al., 2020). Again, exposure of newborns to the maternal vaginal microbiota is considered a source of seeding of the infant gut microbiota (Witkin and Linhares, 2017). However, although dominated by bacteria from the genus *Lactobacillus*, the maternal vaginal microbiota was found to play a minor role in seeding infant stool microbiota. No significant difference was observed in the prevalence or abundance of *Lactobacillus* between babies delivered vaginally or by cesarean section (Shao et al., 2019). It has been increasingly considered that the maternal gastrointestinal tract is the principal source of microorganisms in breast milk (York, 2018), and there is a consensus that breast milk acts as an intermediary for the transfer of functionally important bacteria from mother to infant, especially for potential probiotic bacteria, such as members of *Lactobacillus* and *Bifidobacterium* that can colonize the infant gut (Martín et al., 2007, 2010; Solís et al., 2010; Mikami et al., 2012; McGuire and McGuire, 2017).

Thus, *Lactobacillus* in breast milk was recognized as one of the key contributors to beneficial effects for maternal–infant health by providing an inoculum of *Lactobacillus* to the infant gut. Currently, the *Lactobacillus* species flora of the vagina, which mainly consists of *L*. *crispatus*, *L*. *vaginalis*, *L*. *iners*, *L*. *gasseri*, and *L*. *jenseni*, is markedly different from that of breast milk and infant feces (Zhou et al., 2004; Ravel et al., 2011; Fettweis et al., 2014; Madhivanan et al., 2015). Based on traditional culturing, the main taxa reported in milk were *L*. *casei*, *L*. *salivarius*, *L*. *plantarum*, *L*. *fermentum*, *L*. *rhamnosus*, and *L*. *gasseri* (Albesharat et al., 2011; Martín et al., 2012; Soto et al., 2014; Ding et al., 2019). In infant feces, the *Lactobacillus* species most frequently isolated and detected were *L*. *brevis*, *L*. *fermentum*, *L*. *reuteri*, *L*. *rhamnosus*, and *L*. *plantarum* (Solís et al., 2010; Martín et al., 2012; Jost et al., 2014; Murphy et al., 2017). Overall, the diversity and number of *Lactobacillus* species in breast milk and infant feces varies somewhat with different geographical locations and nationalities. In contrast to those in breast milk and infant feces, more *Lactobacillus* species have been reported in adult feces, but there is considerable variability in the number and species of lactobacilli between individuals (Albesharat et al., 2011; Rossi et al., 2016). Currently, the species composition of *Lactobacillus* in the microbiome of different ecological niches of the human body has been well documented (Rossi et al., 2016; Witkin and Linhares, 2017; George et al., 2018; Ding et al., 2019), yet the cooccurrence of *Lactobacillus* among breast milk, the maternal gut and the infant gut is still only partially understood.

To date, metataxonomics (16S rRNA amplicon analysis) is viewed as a more sensitive and less biased analytical method than culture-dependent methods, allowing a more comprehensive evaluation of the human microbiota (Jost et al., 2013, 2014; Knight et al., 2017; Zuñiga et al., 2017). However, most of the studies performed by sequencing the 16S rRNA gene are generally informative only at the genus level, and accurate identification of bacterial profiles, including the genus *Lactobacillus*, is conducted at the species level. Of note, multiple recent studies have confirmed that vertical transmission of the maternal gut *Bifidobacterium* species to the infant occurs shortly after birth, even showing the sharing of specific strains of *Bifidobacterium* species between microbiomes in the mother-breastmilk-infant triad (Mikami et al., 2012; Jost et al., 2014; Milani et al., 2015; Peirotén et al., 2018). *Lactobacillus*, an important intestinal symbiotic bacterium, in contrast to *Bifidobacterium*, has received less attention to date. In particular, it is not clear how the *Lactobacillus* community from the maternal gut or breast milk progressively transmits to the infant gut during infancy; which of these bacteria will end up colonizing the infant gut is still only partially understood.

China is a developing country, of which the western part is relatively economically underdeveloped but rather culturally diverse. Xinjiang, China, is a multi-ethnic region with a considerable tract of land, in which Uighurs have formed a unique food and marriage tradition and rarely intermarry with other ethnic groups. Therefore, the very diverse microbiomes of local people offer them a rich source of beneficial microbes. In the present study, a comparative analysis of stool and breast milk microbiota in a small yet very homogeneous cohort of 25 healthy mother–infant pairs in Kashgar, northwestern China (*n* = 25, infant age from 7 days to 2 years), was investigated using high-throughput sequencing technologies of the 16S ribosomal RNA gene. In addition, from the perspective of *Lactobacillus* species shared by the mothers and infants, we isolated *Lactobacillu*s strains from the mother-breastmilk-infant triad to try to develop potential probiotic combinations. The purpose of our study was to determine the composition and concordance of *Lactobacillus* populations in breast milk and maternal and infant feces among mother–infant pairs and to identify, as much as possible at the species level, which *Lactobacillus* phylotypes mothers and infants share through breastfeeding in a small yet very homogeneous cohort. We believe that our work will provide a theoretical basis for the development of synthetic probiotics tailored to specific populations.

## Materials and Methods

### Subjects, Study Design, and Sample Collection

Our team recruited 25 mothers and their full-term infants from December 20 to December 24, 2017, at Kashgar, Xinjiang, China. Mother–infant pairs were recruited using the following inclusion criteria: (1) healthy, (2) exclusive or full breastfeeding to at least 4 months post-partum (3) vaginal delivery (≥ 37 weeks gestation), and (4) no antibiotics or probiotic exposure during the pregnancy, intrapartum, and/or postnatal periods. This study was conducted according to the guidelines of the Declaration of Helsinki. Meanwhile, all procedures involving human subjects were then adopted by the Ethics Committee of the First Affiliated Hospital, Shihezi University School of Medicine (2017-117-01). Before the start of the study, written informed consent was issued by all adult participants and all parents or guardians of participating infants.

For breast milk samples, the nipples and areola were cleaned with soap and sterile water, and the first few drops of breast milk were discarded. Then, milk samples (3–5 mL) were collected into sterile collection tubes filled with nitrogen gas. Approximately 5–10 g of fresh fecal samples were collected from the fecal middens of the participants and stored in an anaerobic collection tube filled with nitrogen gas. After collection, the sampling tube was sealed with sealing film and labeled. All the collected samples were temporarily stored in a 4°C car refrigerator and transported back to the laboratory within 4 h. After being transported back to the laboratory, a subsample of breast milk (2–3 mL) and fecal samples (3–5 g) was immediately plated onto de Man, Rogosa, Sharpe (Difco) agar supplemented with 0.05% L-cysteine hydrochloride (Sigma) (MRS-Cys) and *Lactobacillus*-Selective Agar (LBS Agar) plates for culture analysis within 24 h, and the remaining amount was stored at −80°C until DNA extraction.

### Total DNA Extraction and High-Throughput Sequencing

To extract DNA from breast milk samples, we modified the methods of Sakwinska et al. (2016). Briefly, breast milk (1 mL) was taken and centrifuged at 12,000 rpm for 10 min at 4°C. The precipitate was resuspended in 200 μL of Tris-EDTA buffer and treated with 10 μL of lysozyme (50 mg/mL) and 5 μL of DNase-free RNase (20 mg/mL) at 37°C for 30 min. Then, 25 mg of glass beads (10 μm) was added to the solution and treated for 1 min at a speed of 5.5 times per second through three bead-beating steps in a FastPrep instrument (MPBiomedicals, Irvine, CA, United States). After instantaneous centrifugation for 1 min, the supernatant was collected, 20 μL of proteinase K was added, the solution was incubated in a water bath at 56°C for 20 min, 200 μL of GB buffer solution was added, the solution was incubated in a water bath at 65°C for 10 min, and 200 μL of anhydrous ethanol was added. The remaining steps were performed using a Blood gDNA Miniprep Kit (Tiangen, Beijing, CHN) according to the manufacturer’s instructions. Total DNA was extracted from 0.5 g of feces using a Magnetic Soil And Stool DNA Kit (Tiangen, Beijing, CHN) according to the manufacturer’s instructions, with the addition of a bead-beating step (320s × 2). The quantity and purity of extracted DNA were examined by NanoDrop 2000 ultraviolet microspectrophotometer (Thermo Fisher Scientific, United Kingdom) measurement of the OD260/280 ratio. Then, the DNA solution was stored at −20°C until the sample was delivered.

Of the qualified DNA samples, the V4–V5 hypervariable regions of the 16S rRNA gene were amplified and sequenced on an Illumina MiSeq platform by Shanghai Personal Biotechnology Co., Ltd^1^.

### Bioinformatics and Statistical Analysis

16S rRNA gene amplicon sequences were processed through a pipeline combining USEARCH v10.0 (Edgar, 2010) and Quantitative Insights into the Microbial Ecology pipeline (QIIME, version 1.9.1)^2^ (Caporaso et al., 2010). High-quality sequences were subjected to denoising (error-corrected), and chimeras were removed to generate ZOTUs using UNOISE3 (Edgar, 2017). The representative sequence for each ZOTU was screened for further annotation. Then, the representative sequences were employed to annotate taxonomic information using the Ribosomal Database Project (RDP) classifier trained on the Greengenes database v13.8 (Wang et al., 2007)^3^.

For *Lactobacillus* species-level ZOTUs, representative sequences of each ZOTU at information level 7 were identified by using the Basic Local Alignment Search Tool (BLAST) v2.2.29+ against the NCBI 16S Microbial database, with the threshold for sequence identity set at 97% and the *e*-value at 0 (Li et al., 2017). The raw sequence files supporting the findings of this article are available in the NCBI Sequence Read Archive under the BioProject ID PRJNA659245.

Based on the Chao1 and Shannon index (Chao and Shen, 2003), the abundance and diversity of the *Lactobacillus* populations were evaluated, and the differences in different groups were determined by one-way analysis of variance (ANOVA). The beta diversity of the *Lactobacillus* population between different samples was compared using PCoA based on weighted and unweighted UniFrac phylogenetic distances. The function “adonis” of the vegan package was used to test the significance of separation by permutation multivariate analysis of variance (PERMANOVA). Then, the *p*-value of multiple comparisons was corrected by the Benjamini–Hochberg method. *P* < 0.05 was considered statistically significant. Linear discriminant analysis (LDA) effect size (LEfSe) was carried out by using the R package dplyr and open reference strategy^4^ (Zhang et al., 2018). The cooccurrence network was determined using the Spearman correlation coefficient. All figures were made by GraphPad Prism v8.0 (GraphPad Software, San Diego, CA) and R software (v3.6.2) (Robinson and Oshlack, 2010).

We used Phylogenetic Investigation of Communities by Reconstruction of Unobserved States 2 (PICRUSt2) v.2.1.3-b software for population phylogenetic studies (Douglas et al., 2020), 16S rRNA marker gene sequences were used to predict metagenomic function, and the functional composition of the genome was predicted by referring to class 1 and class 2 functional gene classes of the Kyoto Encyclopedia of Genes and Genomes (KEGG). R software (v3.6.2) and STAMP v2.1.3 (Parks et al., 2014) were used for statistical analysis of functional maps.

### Recovery of *Lactobacilli* on Selective Media

To improve the diversity of strain separation, the samples were separated by the direct dilution and plate spreading method. The samples were serially diluted with sterile saline supplemented with 0.5% L-cysteine hydrochloride (Sigma). The dilution suspension (100 μL) was spread plated onto de Man, Rogosa, Sharpe (Difco) agar supplemented with 0.05% L-cysteine hydrochloride (Sigma) (MRS-Cys) (Murphy et al., 2017) and *Lactobacillus-*Selective Agar (LBS Agar) (Beasley et al., 2004). Each gradient dilution was spread on two plates and cultured at 37°C for 24–48 h under anaerobic conditions. According to the morphology, size, color, observation characteristics of liquid culture, and morphology of bacteria under a microscope, 10–15 colonies were selected from each plate, and 35–45 colonies were obtained from each sample. After three culture passages, Gram staining and catalase production experiments were performed for preliminary identification of suspected *Lactobacillus* (Klayraung and Okonogi, 2009). Storage of a pure culture of the strain was performed by adding 20% sterilized glycerol and placement at −80°C.

### Genomic DNA Extraction and DNA Fingerprinting

Genomic DNA was extracted from each of the above fresh colonies as described by Duckchul Park, with slight modifications (Park, 2007). Next, unidentified gram-positive rod-shaped lactic acid bacteria (LAB) isolates from all samples were initially screened and grouped using repetitive PCR (rep-PCR) fingerprints for cost-effective speciation and typing. Rep-PCR was performed using the BOXA1R (5′-CTAC GGCAAGGCGACGCTGACG-3′) primer with its optimal PCR program (Lee et al., 2012). PCR amplifications were performed in a gradient PCR apparatus (Sensoquest Labcycler, GER).

The amplified products were analyzed by horizontal electrophoresis on a 1.5% (w/v) agarose gel in 0.5 × Tris-acetate-EDTA (TAE) buffer for 4 h. The results were observed and photographed in an ultraviolet gel imager (QUANTUM ST5 Vilber Lourmat, France). The comparison of BOX-PCR banding patterns was performed using the computer software package GelCompar II v6.0 (Applied Math, Sint-Martens-Latem, Belgium). The Pearson similarity coefficient was calculated, and the unweighted pair group method with arithmetic mean (UPGMA) was used for grouping. Then, at least one representative strain was selected from each group for further sequencing analysis (Supplementary Figure S3).

### Identification of the Bacterial Isolates

The isolates were identified at the species/subspecies level through PCR sequencing of the 16S rRNA gene by using the forward primer 27F (5′-AGAGTTTGATCCTGGCTCAG-3′) and the reverse primer 1492R (5′-CTACGGCTACCTTGTTACGA-3′) and sequenced at Shanghai Shenggong (Shanghai, CHN) (DeLong, 1992). The obtained sequence was submitted to the GenBank database, and sequence homology analysis was performed by BLAST^5^ to compare the similarity between the test strain and the corresponding sequence of the known lactic acid bacteria. A phylogenetic tree was constructed using the *p*-distances and Kimura-2 parameter distances of MEGA 6.0 software and subjected to 1,000 bootstraps tests (Tamura et al., 2013).

## Results

### Study Population

We enrolled 25 mother–infant pairs. At the initial study visit, we interviewed participants on some of the basic clinical data, including date of birth, height, weight, infant sex, and infant feeding characteristics. The clinical characteristics and demographic data of the mothers and infants are reported in Table 1.

**TABLE 1:** Clinical and demographic characteristics of the mothers and infants in the study population.

Characteristics and demographic data	Values or No. (%)
**Infant sex**	
Male	15 (60)
Female	10 (40)
**Maternal BMI condition**	
Normal (18.5–23.9)	16 (64)
Slightly Fat (24.0–26.9)	4 (16)
Obesity (27–29.9)	3 (12)
Severe Obesity (≥ 30)	1 (4)
Unknown	1 (4)
**Infant age at specimen collection (days)**	
0–180	8 (32)
181–365	8 (32)
366–720	9 (36)
**Breast-feeding**	
Exclusively	9 (36)
Partial feeding	16 (64)
Infant weight (g)	1.58 ± 0.04*
Infant length (cm)	58.50 ± 9.63*
Infant age (days)	312.04 ± 191.71*

*Mean ± SD.

### 16S rRNA Gene Illumina Sequencing of Human Milk, Maternal, and Infant Fecal Samples

16S rRNA gene Illumina sequencing performed on total DNA extracted from breast milk and fecal samples yielded a total of 2,427,427 quality-filtered, taxonomically classifiable 16S rRNA gene sequence reads. According to the rarefaction curves obtained by Shannon and Chao1 metrics, reads with a mean relative abundance less than 0.01% were removed, and the remaining reads were clustered into 1,936 ZOTUs. Among them, a total of 23 phyla and 73 *Lactobacillus* ZOTUs were detected.

Firmicutes, Proteobacteria, Bacteroidetes, and Actinobacteria were the four major phyla in all three ecosystems (Table 2). The average relative abundance of Proteobacteria detected in breast milk samples harbored higher levels compared to that in maternal and infant fecal samples. The average relative abundances of Bacteroidetes in maternal fecal (MF) samples was significantly higher than that in both breast milk (BM) and infant fecal (IF) samples, while the average relative abundances Firmicutes and Actinobacteria in infant fecal samples were significantly higher than in the other two groups. The majority of *Lactobacillales* taxa belonged to *Lactobacillaceae*, *Streptococcaceae*, and *Enterococcaceae*. As one of the important probiotics in the infant gut, *Lactobacillus* has received abundant attention from researchers. The average relative abundances of the *Lactobacillus* genus in breast milk, maternal and infant fecal samples was 5.56, 7.07, and 14.34%, respectively.

**TABLE 2:** Average relative abundance of Illumina reads attributed to the main phyla, to the orders *Lactobacillales*, and the genera *Lactobacillus*.

Phylum/order/genus	Breast milks (BM)	Maternal feces (MF)	Infant feces (IF)
Firmicutes	40.00%	57.66%	60.06%
*Bacilli*	37.54%	24.84%	39.57%
*Lactobacillales*	29.56%	21.87%	38.61%
*Lactobacillaceae*	5.59%	7.09%	14.34%
*Lactobacillus*	5.56%	7.07%	14.34%
*Streptococcaceae*	19.27%	11.93%	23.34%
*Enterococcaceae*	2.48%	1.23%	0.24%
Actinobacteria	2.75%	6.02%	9.17%
Bacteroidetes	4.03%	8.71%	6.53%
Proteobacteria	45.79%	20.25%	20.22%

**FIGURE 1:**
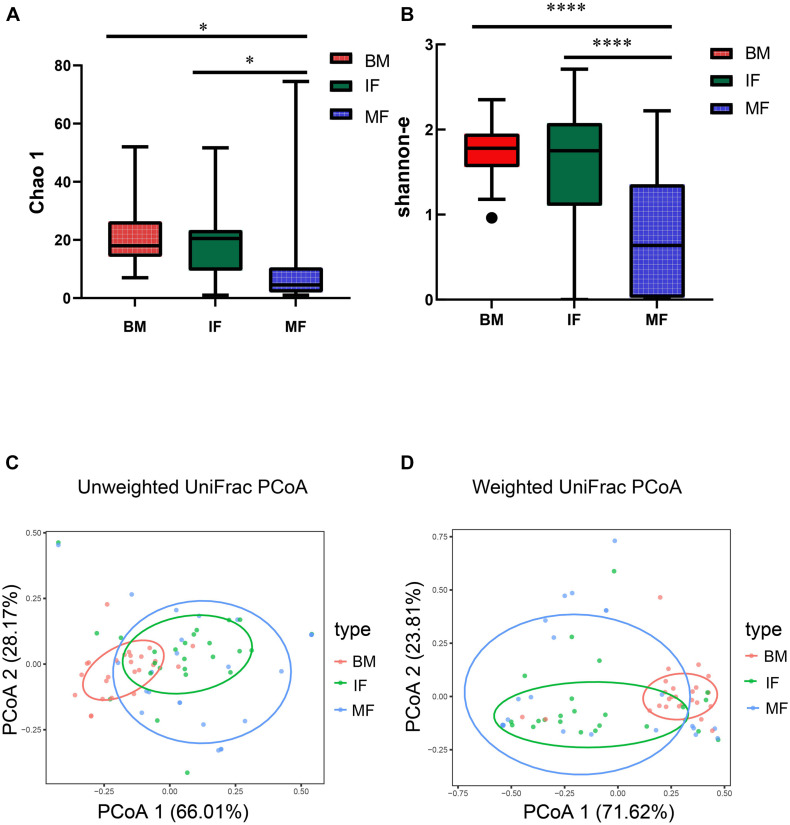
The diversity of *Lactobacillus* populations in breast milk (BM), infant feces (IF), and maternal feces (MF). Box and whiskers distribution of the Chao 1 **(A)** and Shannon α-diversity index **(B)**, calculated for mother’s milk (red), infant feces (green), and maternal feces (blue). These data were analyzed by one-way ANOVA with Tukey–Kramer multiple comparisons test. Principal coordinates analysis (PCoA) of sample clustering results with an unweighted weighted UniFrac distance matrix **(C)** and a UniFrac distance matrix **(D)**. Each point represents a sample (*n* = 75), colored by ecosystem: mother’s milk (red), infant feces (green), and maternal feces (blue). Ellipses represent a 95% confidence interval around the cluster centroid. In both PCoA first and second principal components (PCo1 and PCo2) are plotted. The percentage of variance in the dataset explained by each axis is reported. Statistical analysis was performed using the permutational multivariate analysis of variance (PERMANOVA). In **(A,B)**, *indicates *p* < 0.05, ****indicates *p* < 0.0001.

We further retained 73 *Lactobacillus* ZOTUs for diversity analysis. *Lactobacillus* community richness and diversity were evaluated by the Chao 1 estimator (Figure 1A) and the Shannon index (Figure 1B), separately. The Chao 1 and the Shannon indexes in the BM group and IF group were significantly greater than those in the MF group. There was a significant difference between group BM and MF (Chao 1, *p* < 0.05; Shannon, *p* < 0.0001) or between group IF and MF (Chao 1, *p* < 0.05; Shannon, *p* < 0.0001) but no significant difference between group BM and MF (Chao 1, *p* > 0.05; Shannon, *p* > 0.05). These observations suggested that *Lactobacillus* populations in breast milk and infant feces were more complex than those in maternal feces. The *Lactobacillus* populations between maternal and infant samples were compared by unweighted UniFrac (Figure 1C) and weighted UniFrac (Figure 1D) phylogenetic distances. The results showed that breast milk and fecal samples partially overlapped, while maternal and infant fecal samples largely overlapped. Overall, the infant fecal samples were more similar in the composition of *Lactobacillus* to the maternal fecal samples than to the breast milk samples. PERMANOVA indicated there is significant difference between group BM and MF (unweighted UniFrac, *p* < 0.001; weighted UniFrac, *p* < 0.001) and between group BM and IF (unweighted UniFrac, *p* < 0.001; weighted UniFrac, *p* < 0.001) but no significant difference between group IF and MF (unweighted UniFrac, *p* > 0.05; weighted UniFrac, *p* > 0.05).

To more clearly describe the composition of *Lactobacillus* populations between the mothers and infants, the representative sequences of the *Lactobacillus* ZOTUs were used for homology searching by NCBI BLAST^6^ to obtain more accurate taxonomy results. Taxonomic and phylogenetic information on the ZOTUs is provided in Supplementary Table S1. Except for 15 ZOTUs that could not be identified at specific levels, the remaining ZOTUs were classified into 18 *Lactobacillus* species or species groups. A phylogenetic tree of these *Lactobacillus* ZOTUs was constructed using MEGA 6.0 (Tamura et al., 2013) and the Interactive Tree Of Life (iTOL) (Letunic and Bork, 2016). The majority of the described species branched within the groups were defined by Salvetti et al. (2012). The average relative abundance of several ZOTUs, which were classified as *L*. *ruminis*, *L*. *mucosae*, and *L*. *gasseri*-*johnsonii* in the three ecosystems, was very high. Some ZOTUs only showed a large average relative abundances in breast milk and/or infant feces, while the average relative abundances of all ZOTUs in maternal feces was low (Supplementary Figure S1).

At the species level, a total of 15 *Lactobacillus* species were detected in at least one sample. *L*. *mucosae* and *L*. *ruminis* appeared to be predominant in the three ecosystems. However, diverse *Lactobacillus* populations were present in infant feces, breast milk, and maternal feces (Table 3). Identified as biomarkers in the BM group (LDA > 4), *L*. *gasseri*-*johnsonii* (average relative abundances: 20.26%, occurrence rate: 24/25), *L*. *helveticus* (19.90%, 24/25) and *L*. *mucosae* (21.57%, 23/25) were predominant in all breast milk samples. This result indicated that breast milk samples have relatively abundant and diverse species of lactobacilli that have a significant impact on the microflora.

**TABLE 3:** Average relative abundance and occurrence rate of *Lactobacillus* at the species level among breast milk (BM, *n* = 25), maternal feces (MF, *n* = 25), and infant feces (IF, *n* = 25).

Taxa	Average relative abundance			Occurrence rate		
	BM	IF	MF	BM	IF	MF
*L*. *amylovorus*	0	0.002%	0	0	1/25	0
*L*. *brevis*	3.88%	0.04%	0.002%	24/25	3/25	3/25
*L*. *crispatus-acidophilus*	1.51%	0.90%	0.04%	10/25	10/25	2/25
*L*. *delbrueckii*	1.40%	0.84%	5.00%	12/25	4/25	2/25
*L*. *fermentum*	0.98%	0.75%	0	11/25	9/25	1/25
*L*. *gasseri-johnsonii*	20.26%	8.28%	4.21%	24/25	17/25	6/25
*L*. *helveticus*	19.90%	4.62%	3.52%	24/25	15/25	4/25
*L*. *iners*	0.50%	0	0.006%	1/25	0	1/25
*L*. *mucosae*	21.57%	20.09%	13.97%	23/25	19/25	13/25
*L*. *murinus*	3.44%	1.07%	1.88%	1/25	11/25	3/25
*L*. *oris*	6.36%	2.66%	5.18%	22/25	15/25	7/25
*L*. *parabuchneri-kefiri*	3.51%	0.04%	0	16/25	3/25	0
*L*. *reuteri*	0.05%	0.46%	0.37%	1/25	8/25	3/25
*L*. *rhamnosus*	1.08%	1.37%	0.002%	11/25	5/25	1/25
*L*. *ruminis*	8.46%	22.23%	39.77%	24/25	19/25	22/25
*L*. *salivarius*	4.74%	10.39%	11.25%	14/25	19/25	9/25
*L*. *sanfranciscensis*	0.54%	6.01%	11.82%	4/25	9/25	13/25
*L*. *senioris-curieae*	0.34%	1.50%	0.68%	8/25	15/25	5/25
*L*. sp.	1.49%	18.75%	2.29%	13/25	20/25	8/25

**FIGURE 2:**
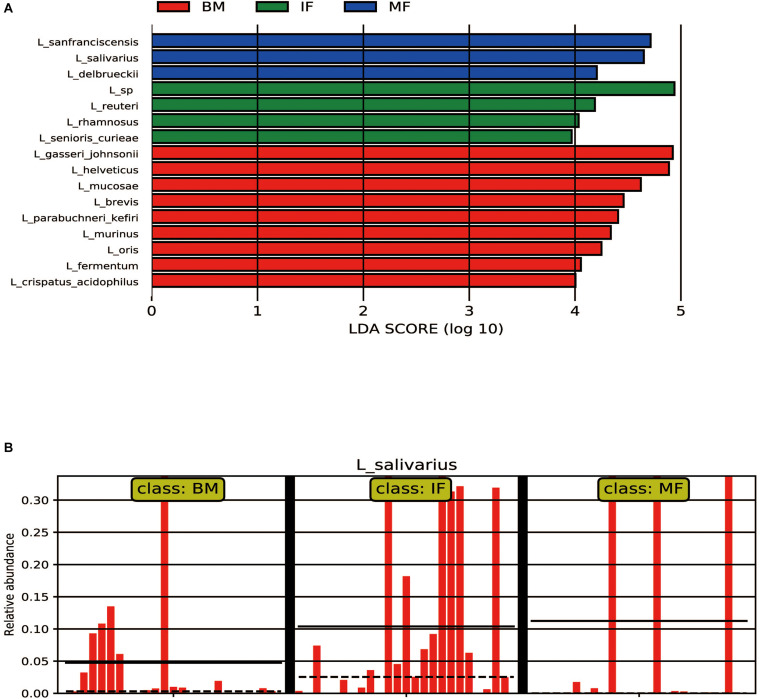
LEfSe analysis of microbiota. **(A)** LEFSe plots showing differences between the maternal and infant lactobacilli of participants. The histograms show the linear discriminant analysis (LDA) scores of bacteriome at the specie level. The length of the histogram represents the LDA score. Breast milk samples (BM) have a higher relative abundance of the taxa shown in red, infant fecal samples (IF) have a higher relative abundance of the taxa shown in green, while maternal fecal samples (MF) have a higher relative abundance of the taxa shown in blue. **(B)** The relative abundance of *L. salivarius* among the BM group (*n* = 25), IF group (*n* = 25), and MF group (*n* = 25).

Regarding the infant fecal samples, *L*. *ruminis* (22.23%, 19/25), *L*. *mucosae* (20.09%, 19/25), and *L*. *salivarius* (10.39%, 19/25) were most prevalent. The average relative abundances of *L*. *seniors*-*curiae* in the IF group was not as high as that of the prevalent species (1.5%, 18/25) but was significantly different than that in the BM and MF groups (LDA > 3). *L*. *sanfranciscensis* (11.82%, 13/25) was identified as a biomarker in the MF group (LDA > 4), which was dominant in maternal feces. Interestingly, although the LDA value of *L*. *salivarius* was high (LDA > 4) in the MF group, the occurrence rate of *L*. *salivarius* was low. The reason for this phenomenon was that *L*. *salivarius* with more than 30% average relative abundances was observed only in three MF samples. In contrast, the occurrence rate of *L*. *salivarius* in the IF group was 76%, and its average relative abundances was significantly higher than that in the BM group (4.74%) and MF group (0.15%, except for the above three samples) (as shown in Table 3 and Figure 2).

This finding was in agreement with analyses performed at the *Lactobacillus* ZOTU level. The *Lactobacillus* species richness and evenness in infant feces was similar to that in breast milk, while the composition of the dominant species in infant feces was similar to that in maternal feces.

**FIGURE 3:**
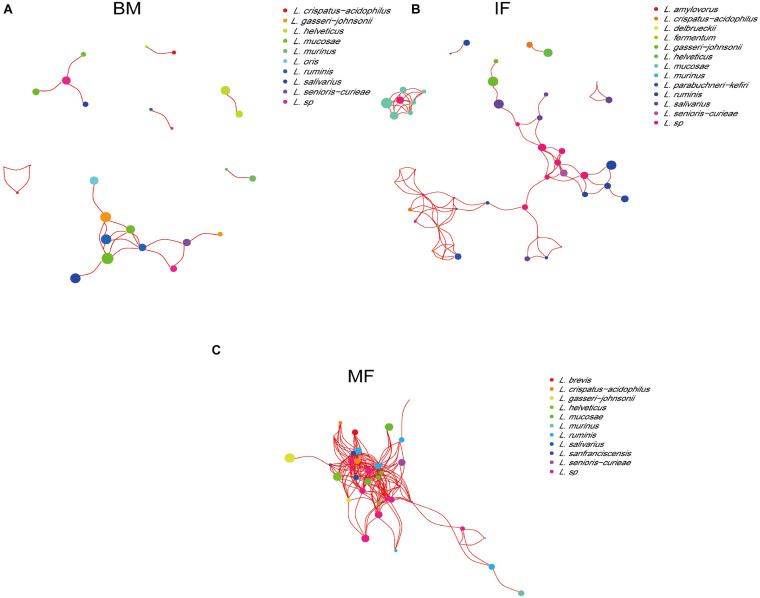
The co-occurrence networks of lactobacilli based on 16S rRNA MiSeq Illumina sequences among the breast milk **(A)**, infant fecal samples **(B)**, and maternal fecal samples **(C)**. Nodes and node sizes represent *Lactobacillus* species and their relative abundances, respectively. Red lines represent positive correlations between the bacteria.

A network analysis of the lactobacilli at the species level based on the Spearman correlation coefficient was used to detect cooccurrence patterns (Figure 3). The analysis of cooccurrence networks showed that the topology of the *Lactobacillus* network in breast milk was the simplest, which might be caused by a large amount of lactose and other nutrients in breast milk being reproduced and utilized by different *Lactobacillus* species. In contrast, the *Lactobacillus* network topology of maternal fecal samples was the most complex, which might be due to the limited nutrient requirements among the lactobacilli. Playing a certain core role in breast milk, *L*. *mucosae* showed relatively high abundance, but there was no cooccurrence relationship with other lactobacilli. *L*. *ruminis* was the core species with the highest relative abundance and positive correlation with other species in the infant and maternal fecal samples.

The composition and relative abundance of *Lactobacillus* in each pair of breast milk and fecal samples as the infant aged is shown in Supplementary Figure S2, to further elucidate the distinct distributions and cooccurrence patterns of *Lactobacillus* between mother and their infants. The *Lactobacillus* communities observed were found to be reasonably complex, and a great deal of variation was observed in most of the samples as the infant aged. The *Lactobacillus* composition of younger infants’ gut (in 7–242 days) was closely related to that of their mothers’ breast milk and/or gut, suggesting that there may be a direct microflora transfer between mothers and infants. Nevertheless, for older infants’ gut (in 242–720 days), the *Lactobacillus* composition was inconsistent with that of their mothers, which may be due to the fact that most of the infants had been introduced to solid food, and obtained microbes from other environments. As outlined in Supplementary Figure S2, *L*. *ruminis* (17 pairs), *L*. *mucosae* (9 pairs), *L*. *helveticus* (4 pairs), *L*. *gasseri*-*johnsonii* (4 pairs), *L*. *oris* (3 pairs), *L*. *salivarius* (3 pairs), *L*. *sanfranciscensis* (1 pair), *L*. *brevis* (1 pair), *L*. *crispatus*-*acidophilus* (1 pair), and *L*. *seniors*-*curiae* (1 pair) were commonly detected in mother–infant pairs. However, *L*. *amylovorus* was detected in only one of the infant fecal samples. Likewise, *L*. *iners* was detected in only one breast milk and one maternal fecal sample. *L*. *reuteri* was detected in only one matched breast milk and infant fecal sample. Additionally, we found that a few specific *Lactobacillus* species cooccurred in the mother-breast milk-infant “triad,” including *L. ruminis*, *L. mucosae*, *L. helveticus*, *L. gasseri-johnsonii*, *L. oris*, and *L. salivarius*.

### Predicted Metagenome Function of Breast Milk, Maternal, and Infant Gut Bacterial Populations

Given the effect of breast milk and the maternal gut microbiota on the infant gut populations’ physiological functions, we used PICRUSt2 analysis to infer the functional metagenomic contents to examined the differences in bacterial functional profiles among the three ecosystems.

**FIGURE 4:**
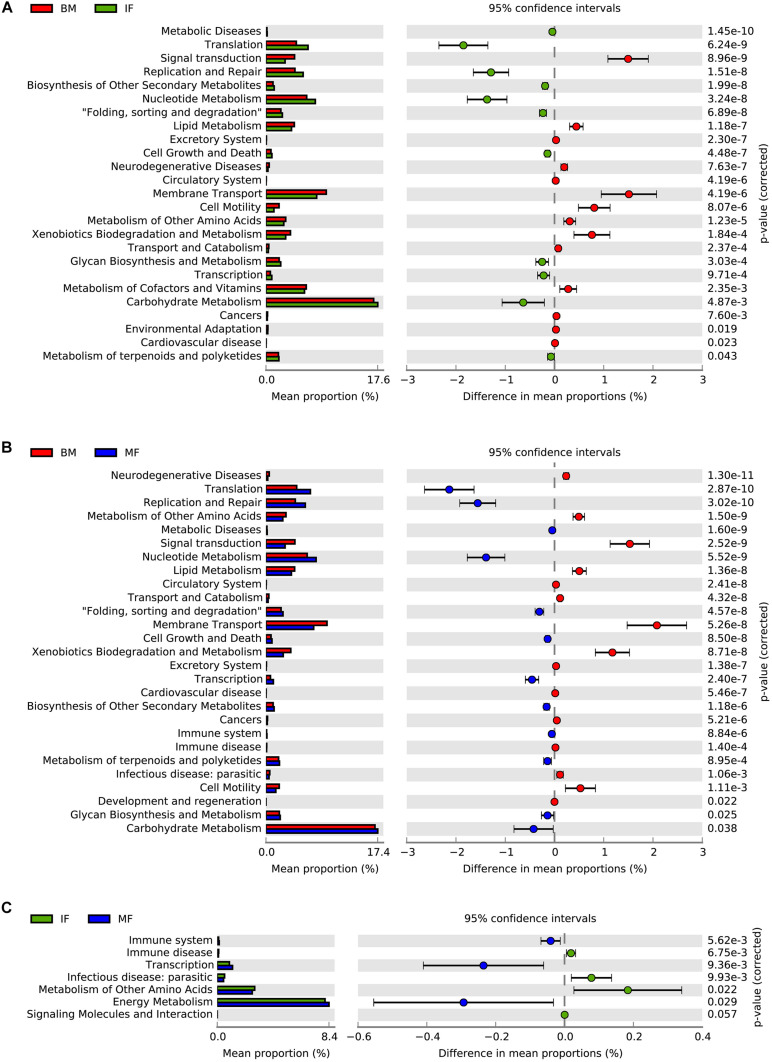
PICURSt2 functions prediction of microbiota in human breast milk (BM), maternal feces (MF), and infant feces (IF). **(A)** Functional differences between BM group and IF group. **(B)** Functional differences between BM group and MF group. **(C)** Functional differences between IF group and MF group.

In the BM and IF groups, the relative abundance of the 25 sub-functions of the secondary function prediction was significantly different. Breast milk contained bacteria with higher predicted abundances in gene families related to signal transduction, lipid metabolism, membrane transport, cell motility, metabolism of other amino acids, xenobiotics biodegradation and metabolism, transport and catabolism, and metabolism of cofactors and vitamins, while the functional genes of replication and repair, translation, nucleotide metabolism, carbohydrate metabolism, transcription, and glycan biosynthesis and metabolism were significantly enriched in the IF group (Figure 4A, *p* < 0.05). There were considerable differences in the relative abundance of 27 subfunctions between the MF and BM groups. In the MF group, functional genes such as translation, replication and repair, nucleotide metabolism, carbohydrate metabolism, glycan biosynthesis and metabolism, and glycan biosynthesis and metabolism were enriched, while in the BM group, functional genes such as metabolism of other amino acids, lipid metabolism, membrane transport, xenobiotics biodegradation and metabolism, and cell motility were significantly enriched (Figure 4B, *p* < 0.05). In the BM and MF groups, the functional genes related to immune disease, infectious disease: parasitic, and metabolism of other amino acids were significantly more abundant than in the IF group, while in the MF group, functional genes including the immune system, transcription, and energy metabolism had a higher abundance (Figure 4C, *p* < 0.05).

### Culture-Dependent Analysis of *Lactobacillus* Strains in All Samples

**FIGURE 5:**
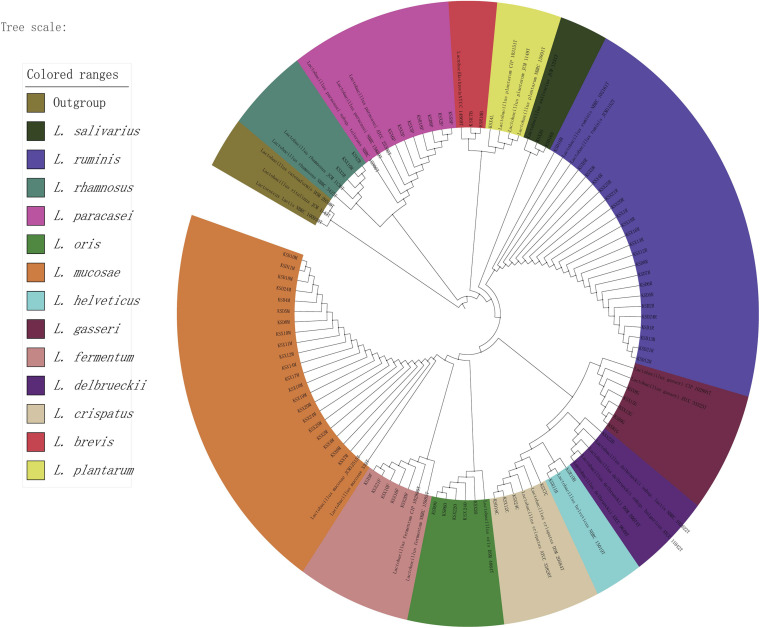
The phylogenetic tree of representative strains of lactobacilli in all samples. The maximum likelihood method was used for the analysis with 1,000 bootstrap repeats.

For breast milk, no colonies could be isolated from > 30% of the samples, and they contained low viable bacterial counts, as detected on the LBS agar medium (0–6.9 log CFU/mL) and the MRS-Cys agar medium (4.48–7.5 log CFU/mL). Conversely, almost all fecal samples readily yielded colonies on MRS-Cys agar medium (5.69–7.22 log CFU/mL) and LBS agar medium (4–7.02 log CFU/mL). After morphological, biochemical and physiological testing, 1,020 isolates that were suspected to be lactobacilli were detected using BoxA1R primers to produce species-specific genomic fingerprints. BOX-PCR strip patterns of the isolates were grouped into well-separated clusters representing individual species. For each BOX-PCR group, 2–3 representative isolates were chosen for partial 16S rRNA gene sequencing. BLAST results showed that almost all *Lactobacillus* strains had a high 16S sequence consistency (97∼100%) with the most recently described relatives in GenBank, indicating that these strains were closely connected with the species already described. Depending on the classification names in GenBank retrieval results, the 16S rRNA sequence obtained in this study divided 539 representative *Lactobacillus* strains from 75 BOX-PCR groups into 13 species. Figure 5 displays the phylogenetic relationships of representative strains of *Lactobacillus* species identified and the closely related, already described species for the genus *Lactobacillus*.

**FIGURE 6:**
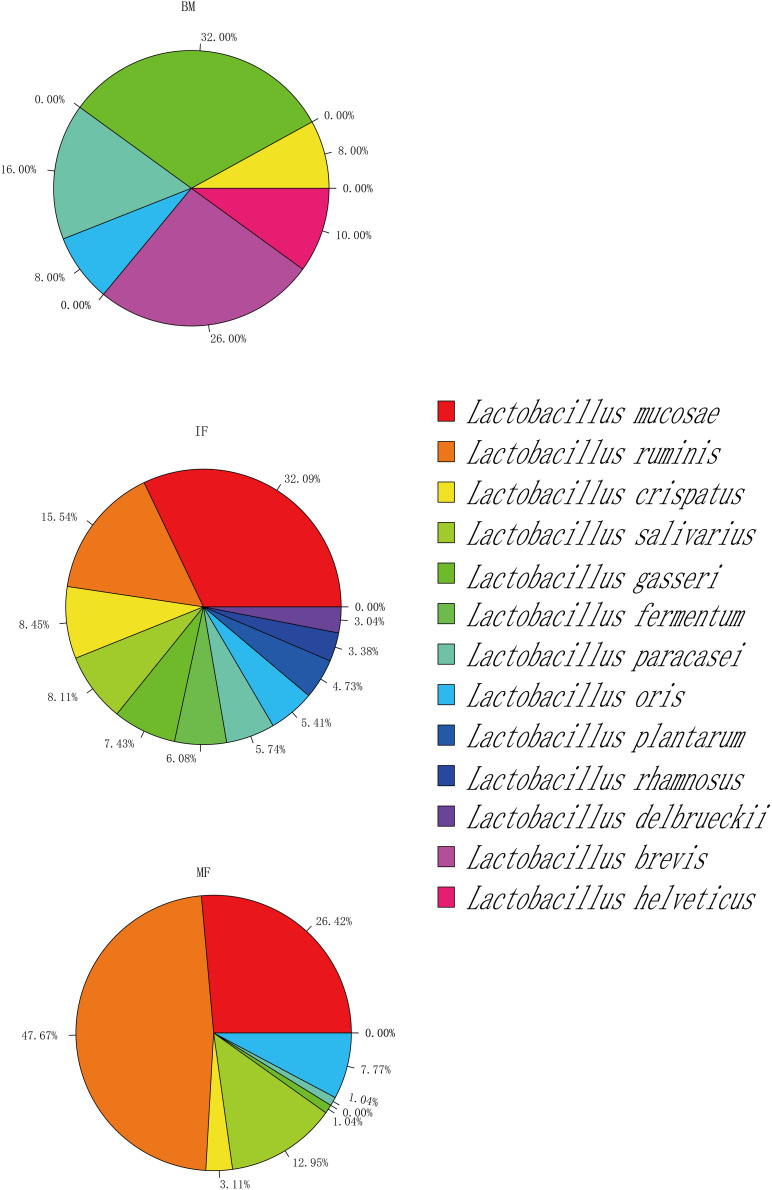
Taxonomy of selected *Lactobacillus* strains isolated from maternal feces (MF, *n* = 25), breast milk (BM, *n* = 25), and infant feces (IF, *n* = 25).

Overall, lactobacilli could be isolated from 13 (52%), 24 (96%), and 23 (92%) samples of breast milk, infant feces and maternal feces, respectively (Supplementary Table S2). Together with the results of Figure 6 and Supplementary Table S3, the isolation frequencies and species compositions of lactobacilli were not uniformly distributed among the three ecosystems. *Lactobacillus* isolates were identified as six species in breast milk samples, representing the following taxa: *L. gasseri* (16 strains), *L. brevis* (13 strains), *L. paracasei* (8 strains), *L. helveticus* (5 strains), *L. crispatus* (4 strains), and *L. oris* (4 strains). There were 9 species isolated in the infant fecal samples, among which the most common species were *L. mucosae* (95 strains), followed by *L. ruminis* (46 strains) and *L. crispatus* (25 strains). Additionally, 193 strains of *Lactobacillus* were isolated in the maternal fecal samples belonging to seven species, including *L. ruminis* (92 strains), *L. mucosae* (51 strains), and *L. salivarius* (25 strains), which were the three species that were isolated most frequently. The viable members of *L. gasseri*, *L. paracasei*, *L. oris*, and *L. crispatus* were commonly detected in the three ecosystems. The phylogenetic structure of breast milk showed a slight dominance of *L. gasseri* (32% isolation frequency) and *L. brevis* (26%), while *L. gasseri* was isolated only from six milk samples, and *L. brevis* was isolated from three milk samples. It is worth noting that strains of *L*. *brevis* and *L*. *helveticus* were isolated only from breast milk samples but not from any fecal samples. *L. ruminis* and *L. mucosae* were predominantly recovered from infant (15.54, 32.09%) and maternal fecal samples (47.67, 26.42%). The isolates of *L*. *fermentum*, *L*. *rhamnosus*, *L*. *delbrueckii*, and *L*. *fermentum* were found only in infant feces samples.

Considering that *L. oris, L*. *paracasei, L. gasseri, L. salivarius, L. crispatus, L. ruminis*, and *L. mucosae* were detected in two and/or three ecosystems simultaneously, these species have the potential to demonstrate vertical mother–infant transfer. *Lactobacillus* species common to at least two of the three ecosystems within mother–infant pairs are listed in Table 4. Interestingly, culturable strains belonging to *L. gasseri* were detected in the same pair of breast milk and maternal fecal samples. *L*. *ruminis* was the bacterial species shared by the most mother–infant feces samples (7 pairs), followed by *L*. *mucosae* (detected in 5 mother–infant pairs). Both *L*. *paracasei* and *L*. *salivarius* were found simultaneously in the maternal feces and infant feces of only one pair. Among the three ecosystems, *L*. *oris* and *L*. *crispatus* existed in unpaired samples.

**TABLE 4:** *Lactobacillus* species shared by mother–infant pairs.

Pairs	Bacterial species													B-I	I-M	B-M
	Lsal	Lgas	Lpcas	Lfer	Lrha	Lbre	Lhel	Loris	Ldel	Lmuc	Lcri	Lrum	Lplan			
N1		+/*										#/*		0	1	1
N2			#	#	#			#		#		*		0	0	0
N3	#/*	#	#		#									0	1	0
N4										#/*	+	#	#	0	1	0
N5			#							*		#/*		0	1	0
N6			#					+		#		#/*		0	1	0
N7	#					+				#	#	*		0	0	0
N8	#	#	#/*							*		*		0	1	0
N9	#	+/#		#				*						1	0	0
N10	#	#		#	#	+	+			#/*		*		0	1	0
N11	*						+	*		#/*				0	1	0
N12										#	#	#/*		0	1	0
N13	#	+/#		#				#				*		1	0	0
N14										#		#/*		0	1	0
N15	#	#										*		0	0	0
N16	*	+										#		0	0	0
N17		+								#		*		0	0	0
N18			+					*		#/*	*	#		0	1	0
N19										#		*		0	0	0
N20								*		#		#/*		0	1	0
N21				#	#							#/*		0	1	0
N22								#	#					0	0	0
N23								+				*		0	0	0
N24		+						#		#/*	#	*		0	1	0
N25										#		#	#	0	0	0

*Lsal, L. salivarius; Lgas, L. gasseri; Lpcas, L. paracasei; Lfer, L. fermentum; Lrha, L. rhamnosus; Lbre, L. brevis; Lhel, L. helveticus; Loris, L. oris; Ldel, L. delbruecki; Lmuc, L. mucosae; Lcri, L. crispatus; Lrum, L. ruminis; Lplan, L. plantarum. B-I, the number of bacterial species shared by the breast milk and infant feces samples. I-M, the number of bacterial species shared by the maternal feces samples and infant feces samples. B-M, the number of bacterial species shared by the breast milk and maternal feces samples. +/#, the bacterial species were present in both the breast milk and infant feces samples. #/^∗^, the bacterial species were present in both the maternal and infant feces samples.+/^∗^, the bacterial species were present in both the breast milk and maternal feces samples. +, the bacterial species was only present in the breast milk sample. #, the bacterial species was only present in the infant feces sample. ^∗^, the bacterial species was only present in the maternal feces sample.*

To further determine whether lactobacilli can be vertically transferred from mother to infant via breast milk, we examined whether those isolates belonging to the same species were isolated from both samples from the same mother–infant pair and showed the identical rep-PCR pattern. Rep-PCR discrimination of *L. gasseri* isolates from mother–infant pairs N1 and N9 revealed different fingerprints for isolates from breast milk and maternal and infant feces. Thus, none of the isolated *L. gasseri* isolates appeared to be transferred from mother to infant via breast milk. Surprisingly, however, *L. mucosae* isolates MF 10–12 and IF 10–6, IF 4–5, and MF 4–11 had the same rep-PCR types. The same was true for the isolates of *L. ruminis* (MF 5–16 and IF 5–13), *L*. *paracasei* (MF 8–15, MF 8–3, and IF 8–17) and *L*. *salivarius* (MF 3–5 and IF 3–12) recovered from infant feces and the associated maternal feces (Supplementary Figure S3). This result may indicate that these isolates belonged to the same strains and may support the hypothesis that lactobacilli can be transmitted vertically from mother to infant.

## Discussion

*Lactobacillus* is one of the first “good” bacteria to colonize the intestinal tract of infants (Clemente et al., 2012). Maternal microorganisms are considered to be the key source of bacteria during the development of gut microflora in infants (York, 2018), and a study has shown that the source of *Lactobacillus* in the infant gut microflora is endogenous (Martín et al., 2003). However, the composition of *Lactobacillus* populations of the mother-breast milk-infant “triad” has not been fully understood until now. Therefore, this study was conducted to investigate the effect of maternal lactobacilli on the occurrence and dynamics of lactobacilli in the infant gut at the species and genus levels.

In our present study, 16S rRNA amplicon analysis showed that the presence of *Lactobacillus* DNA was detectable in all breast milk, maternal, and infant fecal samples. Compared with those of other studies, the occurrence rates and the number of species of *Lactobacillus* that occurred in all samples of this study were higher (Jost et al., 2014; Soto et al., 2014). This may be because 16S rRNA gene amplicon sequence analysis based on the operational taxonomic units (OTUs) clustering algorithm is the most common and cost-effective method for microbial community profiling. However, this clustering algorithm has obvious disadvantages, which reduce the resolution of the phylogeny. In this study, a denoising method was adopted, which removes chimera sequences and possible point errors during PCR and sequencing by denoising the quality-controlled sequences. This method significantly improved the identification rate at the level of species and strains and reduced the proportion of OTU false positives in the results, which is conducive to subsequent experiments and functional analysis (Callahan et al., 2016; Edgar, 2016, 2018; Amir et al., 2017). Combining the results of culture-dependent results, we knew that the occurrence rate of lactobacilli in maternal and infant samples was high. One of the possible explanations for these findings is that our study subjects were volunteers who underwent vaginal deliveries and exclusively breastfed their infants, and the content of *Lactobacillus* in this case was higher than in the case of cesarean section and formula feeding (Yang et al., 2019). A review of existing research shows that the probiotic soluble breast milk oligosaccharides (HMOs) contained in breast milk can promote the growth of *Lactobacillus* and maintain the integrity of the intestinal epithelial barrier function (Borewicz et al., 2019). Infants born by vaginal delivery had an increased colonization rate for *Lactobacillus* spp. compared with that of infants born by elective cesarean delivery at term (Nagpal et al., 2016). Another aspect is that all subjects of the study were located in northwestern China, and their diet is dominated by dairy products. This diet usually leads to more *Lactobacillus* species in breast milk (Ding et al., 2019).

In this study, the Illumina MiSeq sequencing showed that the species richness and diversity of the *Lactobacillus* population in the infant gut were similar to those of the *Lactobacillus* population in breast milk, and the shared phylotypes accounted for a higher proportion of the total abundance of the respective flora. This result was similar to previous related studies (Martín et al., 2007; Murphy et al., 2017). This finding is not surprising because infants have less restrictive stomach conditions than adults, so *Lactobacillus* from breast milk is more likely to colonize the infant gut (Martín et al., 2012). However, the dominant species and the relative abundance of the *Lactobacillus* population of the infant gut were similar to those of the maternal gut. Furthermore, combined with the isolation of live bacteria, it was found that the strains from the maternal gut that colonized the infant had better adaptability.

When the culture-dependent data were compared with the culture-independent data, we found that *L. ruminis* and *L. mucosae* were the most abundant species in the fecal samples. Furthermore, Except for *L*. *amylovorus*, *L*. *fermentum*, *L*. *iners*, *L*. *parabuchneri*-*kefiri*, the DNA of other *Lactobacillus* species or species groups were simultaneously detected in the BM, IF, and MF groups. But, only *L. crispatus*, *L. gasseri*, *L. paracasei*, and *L. oris* were simultaneously isolated from maternal and infant samples based on culture-dependent approaches. Among them, *L. gasseri* and *L. paracasei* were also detected simultaneously in mother-breast milk-infant “triad” samples from Syria (Albesharat et al., 2011). Additionally, *Lactobacillus* DNA was commonly found in breast milk samples, but only 6 *Lactobacillus* species were isolated on the selective medium, namely, *L. crispatus*, *L. paracasei*, *L. gasseri*, *L. oris*, *L. brevis*, and *L. helveticus*, which were isolated and detected in previous studies (Solís et al., 2010; Martín et al., 2012; Mehanna et al., 2013; Soto et al., 2014; Sallam et al., 2015). Additionally, it is worth noting that the DNA of a few species of *Lactobacillus* was detected by culture-independent technology, but the species could not be recovered on the media. This result is expected because the *Lactobacillus* in the maternal and infant samples (especially in breast milk) was suboptimal, and the similar nutritional and environmental requirements between *Bifidobacterium* and *Lactobacillus* hindered the isolation of *Lactobacillus* (Quartieri et al., 2016). Specifically, *L. ruminis*, *L. mucosae, L. crispatus*, *L. delbrueckii*, *L. fermentum*, *L. gasseri*, *L. johnsonii*, *L. paracasei*, *L. plantarum*, *L. reuteri*, *L. rhamnosus*, and *L. salivarius* were the most abundant and resident populations of the human gut. Therefore, they were easy to find and isolate. However, some species, such as *L. murinus*, are considered gut-autochthonous microorganisms. *L. sanfranciscensis* is a transient bacterium in the human gastrointestinal tract. They were of extremely low abundance and were not easily obtainable (Lebeer et al., 2008; Rossi et al., 2016).

We compared the *Lactobacillus* phylotypes in the infant gut with those of their mothers vs. random mothers. We found more shared species-level phylotypes in the correct pairs of the mother–infant samples. It is interesting that Daft et al. (2015) cross-fed mice from two breeds within 48 h of birth and found that the microbiota in the feces of the mice was similar to that of the nursing mothers rather than the biological mothers. In addition, an animal experiment showed the presence of two specially labeled *Lactobacillus* strains in the mammary tissue and milk of pregnant mice by oral administration (de Andrés et al., 2017). This result also means that maternal strains were ecologically more adaptable in infants than non-maternal strains (Ferretti et al., 2018). Based on bacterial culture-dependent technology, *L. gasseri* strains were found in infant feces and breast milk, and *L. ruminis*, *L. mucosae, L. paracasei*, and *L. salivarius* strains were also found in maternal and infant fecal samples. This study further demonstrated that *L. ruminis*, *L. mucosae*, *L*. *paracasei*, and *L*. *salivarius* isolates recovered from between maternal and infant feces were shown to be identical. This finding provided additional support for the hypothesis that *Lactobacillus* from the mother’s breast milk and feces is transferred to the infant gut, that is, *Lactobacillus* is endogenous in mother-to-infant transmission. In particular, *L. gasseri* has been shown to transfer to the infant gut through breast milk (Martín et al., 2012). A study by Pannaraj et al. (2017) found that nearly 40% of fecal bacteria in breastfed infants come from breast milk and areola skin, of which breast milk accounts for nearly 30%. Other sources may include the uterine environment, maternal intestines and vagina, and the external environment (Pannaraj et al., 2017). These findings suggest that the entero-mammary pathway is an important link between the intestinal flora of mothers and infants. Through this route, the maternal intestinal bacteria migrate to the mammary gland, where they are passed on to the infant and participate in the formation of the infant intestinal flora (Rodríguez, 2014). However, the complex mechanism of the entero-mammary pathway is not yet fully understood. It is not clear how the bacteria in the maternal gut interact with immune cells and how they are transported to the breast and evade the defense mechanisms of immune cells, and further research is needed to determine whether there is a specific period for bacterial migration in the entero-mammary route of transmission.

Our predicted metagenomic functional analysis suggested that the maternal gut and breast milk microbiota may have some influence on the physiological function of infants. Among them, there were significantly more functional genes for carbohydrate metabolism and sugar anabolism in the gut of infants. Additionally, it was also found that the relative abundances of *L. mucosae*, *L. reuteri*, and *L. ruminis* in the gut of infants were increased. Studies have shown that *L. mucosae* has a complete glycogen metabolic pathway in the pangenome (Valeriano et al., 2019). *L. reuteri* has a strong ability to make use of maltose, sucrose, raffinose, and (iso)-malto-oligosaccharides (Zhao and Gänzle, 2018). Human-residential *L. ruminis* strains can use a variety of carbohydrates (Wang et al., 2020). Breast milk contains abundant genes related to exogenous biodegradation and metabolism of bacteria, lipid metabolism, cofactor, and vitamin metabolism, amino acid metabolism, etc. These functional genes are involved mainly in the digestion and absorption of fats and proteins. This may be because breast milk has more easily digestible protein and unsaturated fatty acids, which are more conducive to the digestion and absorption of infants.

The health-promoting, nutritional and physiological, microbiological, and immunological effects of lactobacilli have been proven. Furthermore, most of the *Lactobacillus* species isolated in this study are considered to be potential probiotics and have the status of generally recognized as safe (GRAS) and qualified presumption of safety (QPS) by the Food and Drug Administration and the European Food Safety Authority, respectively (Heeney et al., 2018; Zhang et al., 2018). *L. ruminis* and *L. mucosae*, as autochthonous members of the human gut microbiota, are not included in the list of bacteria that can be used in food, but their immunomodulatory, anti-inflammatory, and antioxidant properties have been confirmed by studies (Taweechotipatr et al., 2009; Yu et al., 2017; Ryan et al., 2019). Therefore, this study identified new potential probiotics from local sources as local populations, enabling residents in developing countries to gain commercial promotion and health-promoting opportunities from locally produced probiotic products, thus providing a valuable, healthy and affordable alternative to commercial probiotic products.

## Conclusion

Bacteria that relocate from the birth canal during parturition and from breast milk during lactation affect the colonization of gastrointestinal microorganisms in newborns (Funkhouser and Bordenstein, 2013; Verdu et al., 2015; Martin et al., 2016). In this study, we combined culture-dependent and culture-independent methods to confirm the existence of vertical transmission routes of *Lactobacillus* phylotypes in 25 pairs of mothers and infants and the stability of the occurrence of the *Lactobacillus* population between mothers and infants. Given the data on *Lactobacillus* presented, it still seems non-definitive for the *Lactobacillus* transmission through breast milk, but the similarity proportion of the general BM microbiome may give some context to this pathway. This finding is especially important because infant formula sold on the market is virtually free of microbes after pasteurization, while infants born by cesarean section or without breastfeeding are vulnerable to disease. Therefore, the potential probiotics recovered from local mother–infant pairs will be used to adjust the maternal intestinal environment and microflora tailored to specific populations to remedy the adverse effects of the industrial lifestyle (cesarean section, infant formula milk, and industrial food consumption) on the gut microflora.

The limitation of this study is that there is no in-depth analysis of *Lactobacillus* phylotypes between mothers and infants. For example, an in-depth analysis of breast milk and the maternal gut flora, a correlation analysis of the maternal skin and vaginal flora, and tracking the relationship between development and health status of infant gut *Lactobacillus* populations would further clarify at the strain level how mother-to-infant transmission affects other components of the gut microflora in infants and the importance of the maternal flora in infant gut colonization.

## Data Availability Statement

The datasets presented in this study can be found in online repositories. The names of the repository/repositories and accession number(s) can be found below: https://www.ncbi.nlm.nih.gov/genbank/, MT705748–MT705827; www.ncbi.nlm.nih.gov/, BioProject ID PRJNA659245.

## Ethics Statement

The studies involving human participants were reviewed and approved by the Ethics Committee of the First Affiliated Hospital, Shihezi University School of Medicine (2017-117-01). Written informed consent to participate in this study was provided by the participants’ legal guardian/next of kin.

## Author Contributions

XZ: formal analysis, methodology, validation, and writing—original draft. SM: methodology and investigation. BL: resources. FT: project administration and funding acquisition. WY: conceptualization, c software, and writing—review and editing. YN: conceptualization, supervision, project administration, and funding acquisition. All authors contributed to the article and approved the submitted version.

## Conflict of Interest

The authors declare that the research was conducted in the absence of any commercial or financial relationships that could be construed as a potential conflict of interest.

## Footnotes

1 http://www.personalbio.cn/

2 http://qiime.org/

3 http://greengenes.secondgenome.com

4 http://huttenhower.sph.harvard.edu/galaxy/

5 http://blast.ncbi.nlm.nih.gov/

6 https://blast.ncbi.nlm.nih.gov/BlastAlign.cgi

## Supplementary Material

Supplementary Figure 1 Circular maximum likelihood phylogenetic tree based on ZOTUs representative sequences of Lactobacillus spp. detected in all samples of breast milk (BM), infant feces(IF), and maternal feces(MF). The bars in the outer band represent the average relative abundances of ZOTU.

Supplementary Figure 2 The Lactobacillus community composition and relative abundance in each of 25 mother-infant pairs with different infant ages. Each panel shows relative abundances of the 19 Lactobacillus species and/or species groups, with each bar representing a sample and each colored bar segment representing the relative abundance of the species and/or species group indicated in the colour key below.

Supplementary Figure 3 Rep-PCR fingerprints of Lactobacillus spp. isolated from breast milk (BM), maternal feces(MF) and infant feces(IF) of partial mother-infant dyads, using primer BOXAIR. Isolates highlighted in bold indicated identical Lactobacillus strains which were recovered from at least two of the three ecosystems.

Supplementary Table 1 Lactobacillus species-level zero radius operational classification units identified in all maternal and infant samples.

Supplementary Table 2 Taxonomy of Lactobacillus isolates in all maternal and infant samples based on 16S rRNA gene sequence alignments with Gen Bank (≥97% sequence similarity).

Supplementary Table 3 Occurrence and composition of Lactobacillus species in all maternal and infant samples.
