# Supplementary figures and images for: The Composition and Concordance of Lactobacillus Populations of Infant Gut and the Corresponding Breast-Milk and Maternal Gut
Source: Front Microbiol. 2020 Dec 21;11:597911. doi: 10.3389/fmicb.2020.597911 (PMC7779531; doi:10.3389/fmicb.2020.597911)

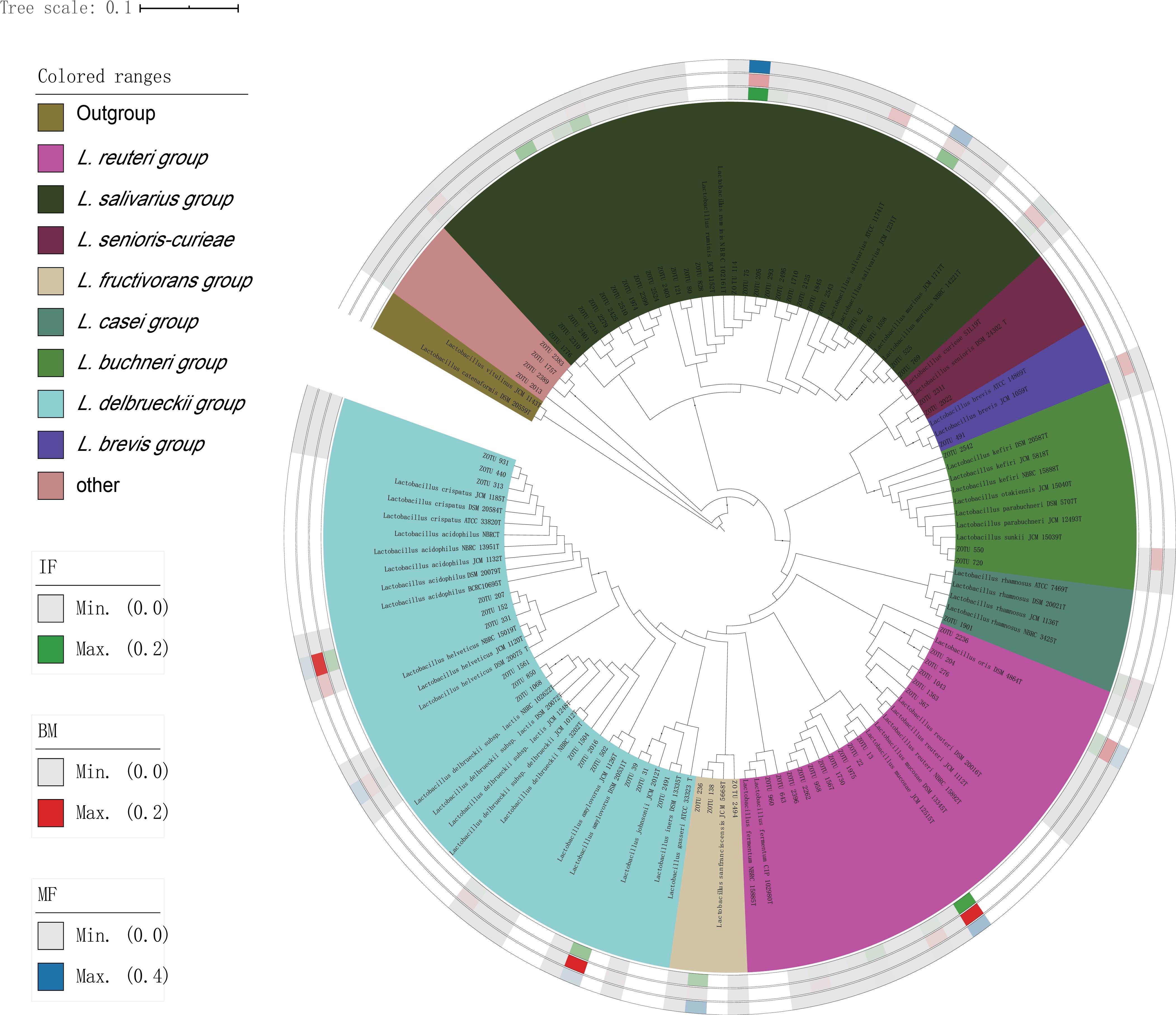

Supplement: Supplementary Figure 1 — Circular maximum likelihood phylogenetic tree based on ZOTUs representative sequences of Lactobacillus spp. detected in all samples of breast milk (BM), infant feces(IF), and maternal feces(MF). The bars in the outer band represent the average relative abundances of ZOTU. [file Image_1.JPEG]

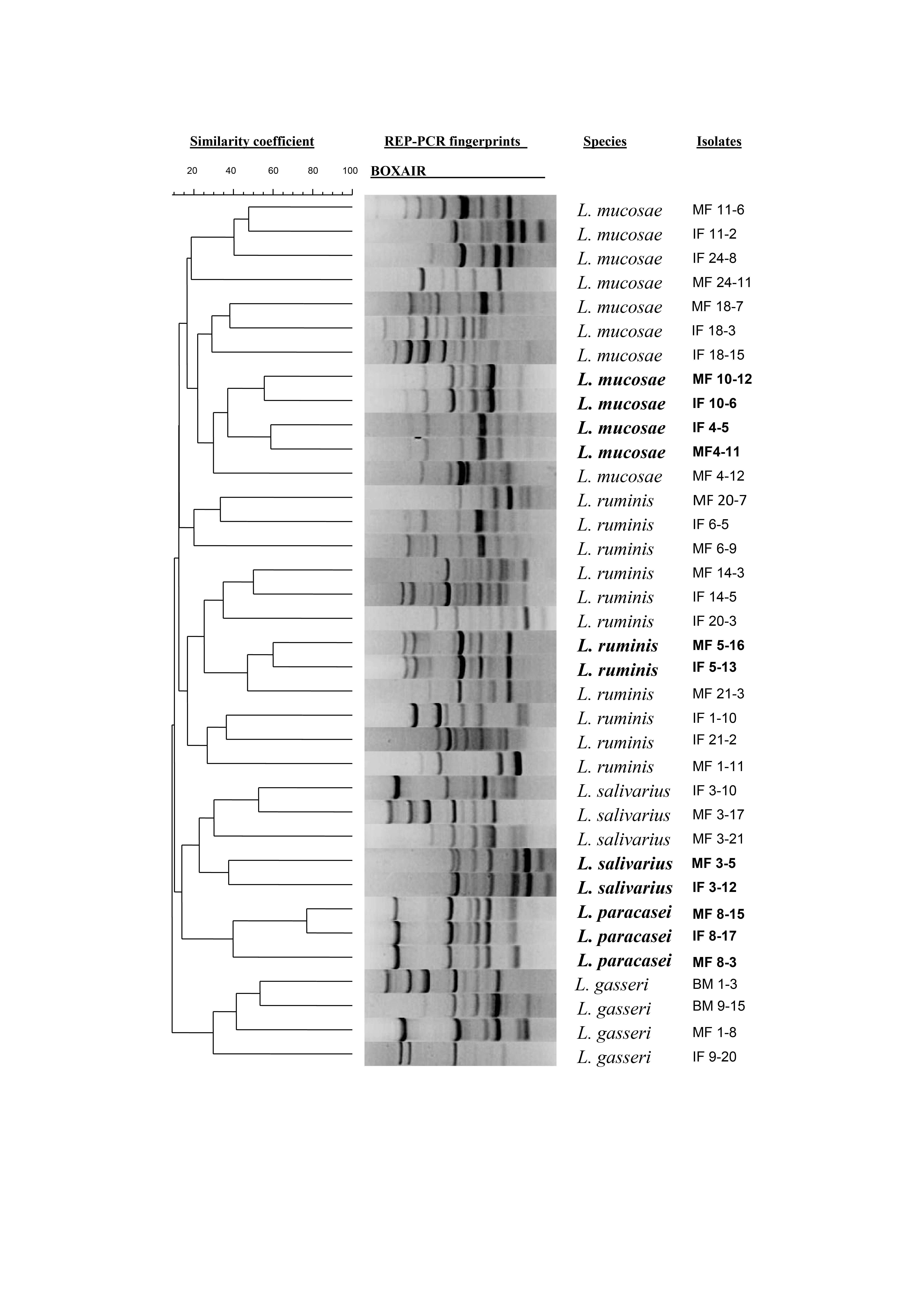

Supplement: Supplementary Figure 3 — Rep-PCR fingerprints of Lactobacillus spp. isolated from breast milk (BM), maternal feces(MF) and infant feces(IF) of partial mother-infant dyads, using primer BOXAIR. Isolates highlighted in bold indicated identical Lactobacillus strains which were recovered from at least two of the three ecosystems. [file Image_3.JPEG]
